# “*V*” aortoplasty of the proximal descending aorta in the elephant trunk procedure

**DOI:** 10.1186/s13019-015-0217-x

**Published:** 2015-01-31

**Authors:** Adrian Kolesar, Boris Bily, Lubomir Spak, Jan Luczy, Panagiotis Artemiou, Frantisek Sabol

**Affiliations:** 1 Clinic of Cardiac Surgery, Eastern Slovak Institute for Cardiovascular Diseases, 8, Ondavska St., 040 11 Kosice, Slovak Republic; 2 Clinic of Angiology, Eastern Slovak Institute for Cardiovascular Diseases, Kosice, Slovak Republic

**Keywords:** Aortic aneurysm, Elephant trunk, Aortoplasty

## Abstract

Complex pathology of the aorta, especially in patients presenting an aneurysm involving the entire aortic arch and proximal descending aorta has been approached in one or two stages. Surgical management of those with an extremely wide diameter of the proximal descending aorta is not yet well defined. The patient in this case was an asymptomatic 47-year-old female with systemic lupus erythematosus (SLE) associated with aneurysm of the ascending aorta, whose aortic arch and descending aorta had presented only overall weakness (examination by inspection and palpation without histological verification). The imaging identified a giant aorta arising at the level of the sinotubular junction (STJ), ending up immediately below the diaphragm. In the first stage she underwent surgical replacement of the entire ascending aorta, aortic arch and proximal part of the descending aorta by combining the elephant trunk with a new type of aortoplasty. In the second stage an endovascular stent graft was inserted into the elephant trunk in the descending aorta. The patient continues to do well 20 months following the repair. In this manuscript type we describe a novel technique of “V” aortoplasty of the proximal descending aorta in order to facilitate the performing of anastomosis between the Dacron graft and aortic aneurysm.

## Background

Complex aortic pathology, especially in patients presenting an aneurysm involving the aortic arch and proximal descending aorta, has been approached in one or two stages [1,2]. In 1983, Borst [3] introduced a significant modification of the two-stage technique – *the elephant trunk procedure*. The largest commercially available aortic Dacron graft is 40 mm, so in cases of very large aortic aneurysmal dimensions, where the elephant trunk procedure is required, it might be technically difficult to perform anastomosis between the Dacron graft and aortic aneurysm.

In this report we describe a novel technique of aortoplasty of the proximal descending aorta in order to facilitate the performing of anastomosis between the Dacron graft and aortic aneurysm.

## Case presentation

### Presentation

A 47-year-old female with SLE having an asymptomatic aortic aneurysm (only overall weakness of aortic wall examined by inspection and palpation without histological verification) was treated with a hybrid and novel procedure. The origin of the aneurysm was at the level of the STJ.

### Investigation

**Figure 1:**
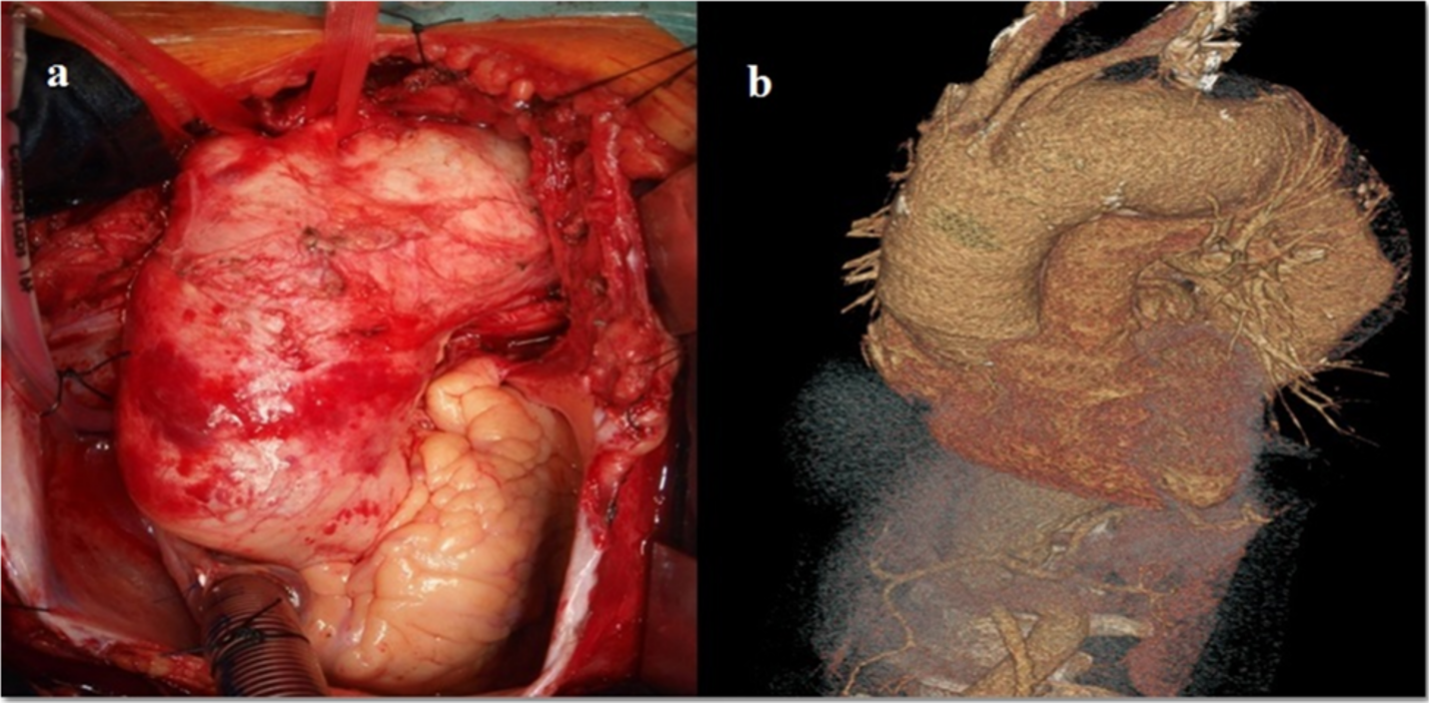
**Preoperative and perioperative images.** Perioperative depiction of the patient's aortic size and anatomy **(a).** The CT scan of the aortic aneurysm **(b)**.

The maximum aortic diameter was 8 cm in the aortic arch extending into the proximal part of the descending aorta, where it extended up to 10 cm (Figure 1a). The preoperative computerized tomography (CT) scan revealed a laminar mural clot and aneurysm morphology (Figure 1b). The preoperative trans-thoracic echocardiographic (TTE) and trans-esophageal echocardiographic (TEE) examination was performed in patient. It revealed saccular aneurysm of ascending aorta (maximal dimension 5.2 cm), which continued to aortic arch (diameter of dilatation 4.6 cm) and included also whole portion of descending thoracic aorta (diameter of dilatation at least 6.3 cm). There was also present laminar mural clot in the lumen of descending thoracic aorta. In the abdominal segment of aorta, there was visible narrowing of dimension to 3.5 cm. Dilatation of aorta finished at the level of truncus coeliacus (diameter of abdominal aorta in this segment was 2.8 cm). Aortic valve had 3 slightly fibrotic changed leaflets without aortic regurgitation (AoR). Diameters of aortic root included aortic ring of 22 mm, aortic bulbus of 42 mm and STJ of 31 mm, respectively. Patient had preserved ejection fraction (EF) of left ventricle of 60%. Additional valve diseases included mild-moderate mitral regurgitation (MR), moderate tricuspid regurgitation (TR) with moderate pulmonary artery hypertension (PAH). Pulmonary artery systolic pressure (PASP) reached 55 mmHg. The patient had a normal coronary angiogram. From associated diseases she had lupus nephritis, neuro-lupus, hypertension and chronic venous insufficiency. She was therefore assessed as an extremely high risk patient with regard to the conventional one-stage procedure.

### Treatment

The hybrid procedure was carried out in two single stages. The first stage included the open surgical approach with replacement of the ascending aorta, aortic arch, and with the performance of proximal V-shape descending aortoplasty. Leaflets of aortic valve were only slightly fibrotic changed and there was not present AoR, so intervention at the level of the aortic valve was not necessary. The procedure was performed through median sternotomy. Arterial cannulation was achieved through the right subclavian artery. Deep hypothermic circulatory arrest at 18 degrees Celsius, with antegrade cerebral perfusion was used during the procedure. Replacement of the ascending aorta with a Dacron graft (size 28) with resuspension of the STJ followed. Next, the replacement of the aortic arch using another Dacron graft (size 34) was performed using *the island technique*. The proximal descending aorta had been exposed with a diameter of 10 cm. In order to facilitate anastomosis between the Dacron graft (size 34) and aortic aneurysm, and due to the disproportional dimensions between the graft and aorta, we accomplished the so-called “*V*” type descending aortoplasty.

**Figure 2:**
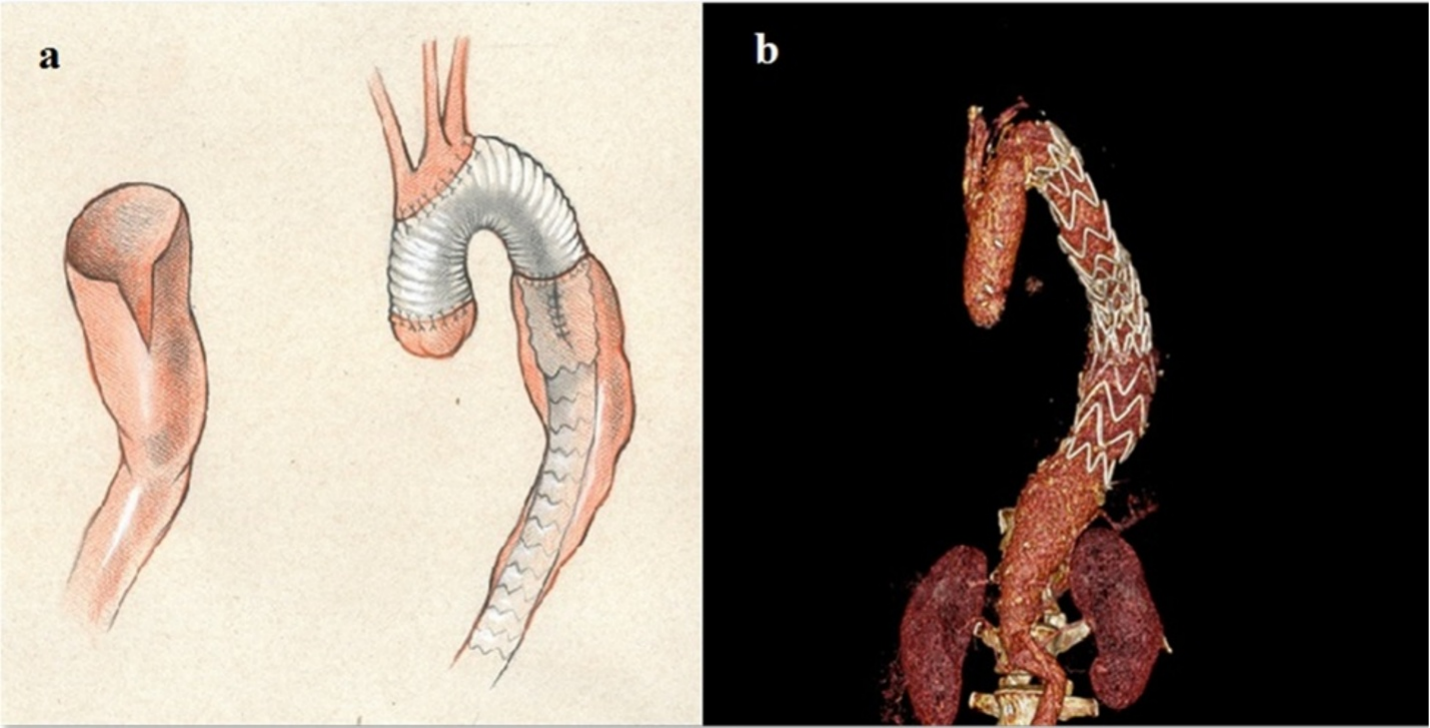
**Animation and postoperative image.** Animation of “*V*” descending aortoplasty (on the anterior wall of the proximal descending aorta a V-shaped aortic wall resection of 5 cm in length and 3 cm in width was done and the defect on the aortic wall was primarily closed with a suture after the elephant trunk had been inserted into the descending aorta **[a]**). Postoperative image of the patient’s final aortic treatment (CT scan **[b]**).

The technique of “*V*” type aortoplasty was as follows: on the anterior wall of the proximal descending aorta, a “*V*” type aortic wall resection of 5 cm in length and 3 cm in width was done and the defect on the aortic wall was primarily closed with a suture after the elephant trunk had been inserted into the descending aorta (Figure 2a).

In the second stage one month later, the patient underwent endovascular stentgraft insertion (Endurant Valiant Captivia VAMF3636C150TE) into the elephant trunk in the descending aorta. The follow-up CT scan revealed total aneurysm sac exclusion without any endoleak (Figure 2b).

## Discussion

Complex aortic pathology, especially in patients having aneurysm involving the aortic arch and proximal descending aorta, has been approached with either a one-stage or two-stage procedure [1,2]. In the latter case, the aortic arch was replaced first, followed by the remaining affected aorta during the second operation 4 to 12 weeks later [1]. Both approaches presented technical challenges; the one-stage procedure due to its long duration and hypothermic circulatory arrest, and the two-stage procedure due to the need of a second operation, which sometimes required another period of circulatory arrest to perform anastomosis to the distal aortic arch. Moreover, the access to the distal aortic arch, usually via the left thoracotomy, was associated with surgical complications related to the densely adherent tissue surrounding the transverse aortic arch prosthesis and vital anatomical structures such as the pulmonary artery, left recurrent laryngeal and vagus nerves, and the esophagus [4].

In 1983, Borst introduced a significant modification to the two-stage technique. In two patients affected by aneurysms involving the ascending aorta, arch and descending aorta, the prosthesis was sutured to the proximal transected descending aorta downstream from the left subclavian artery during the first stage. This had been left, reaching antegradely into the descending aorta over 7 or 8 cm, giving the appearance of an ‘*elephant trunk*’. The second prosthesis was then sutured proximally to the first graft and descending aorta and employed to reconstruct the aortic arch. During the second stage performed a few weeks later, the remaining dilated descending aorta was replaced using the elephant trunk prosthesis for clamping and suturing another graft [3].

Soon thereafter, to avoid the risk of tearing the aorta along the fragile distal suture line during the first stage and to reduce the circulatory arrest time required to suture the additional graft in order to reconstruct the aortic arch, Svensson modified the original Borst technique. He inverted the tubular graft, placed it into the descending aorta, and sutured a double-layer head onto the descending aortic wall. The inner segment could then be retrieved and used for the arch reconstruction as usual, leaving an elephant trunk in the descending aorta. The modification allowed tightening of the distal suture line, a greater surface area between the graft and aortic wall, and reduction of circulatory arrest times, making one of the anastomoses redundant [5].

The presence of a significant interval of mortality between the two stages ranging from 3% to 13%, the fact that only 45% of patients who underwent the first-stage elephant trunk procedure returned for the second-stage completion, and the complications related to the second-stage procedure have convinced some surgeons to perform, whenever possible, the one-stage repair through the clamshell [6-8], trans-mediastinal [9] or left posterolateral thoracotomy approach [10].

However, the longer operation times associated with extensive one-stage or total replacements of the entire aorta [11,12], higher pulmonary complication rates ranging from 15% to 50%, the need to sacrifice both internal mammary arteries, the postoperative pain, and inability to extend resection to the segments downstream from the diaphragm have considerably limited their applicability and acceptance. At present, only one-stage repair is performed in a small number of centers.

To shorten the interim interval between the stages, other surgeons have propagated hybrid approaches with endovascular completion of the first-stage elephant trunk, implanting covered stent grafts in both the antegrade [13] and retrograde fashion [14-16]. This option has been considered particularly appealing, as the elephant trunk thus functions as a landing zone for such stent grafts.

SLE may affect both the central nervous system (CNS) and the peripheral nervous system (PNS). Neuro-lupus is caused by attacking the nervous system via antibodies that bind to nerve cells or the blood vessels that feed them. Another choice represents the interruption of blood flow to nerves. The most common manifestation of neuro-lupus represents cognitive dysfunction. It is characterized by confusion, clouded thinking and impaired memory. Single photon emission computed tomography (SPECT) scans of SLE patients with cognitive dysfunction show abnormalities in blood flow (indicating that the condition may be the result of decreased oxygen delivery to certain parts of the brain). Almost 20% of patients with lupus have migraine-like headaches. A true lupus headache usually requires corticosteroids for treatment [17].

In our case, after a successful replacement of the ascending aorta and aortic arch, the patient underwent a second-stage procedure on postoperative day 20, which involved an endovascular stent graft insertion to the descending aorta where the elephant trunk functioned as a landing zone for the stent graft. As the largest commercially available aortic Dacron graft is 40 mm wide, in cases of very large aortic aneurysmal dimensions where the elephant trunk procedure is required, it might be technically difficult to perform anastomosis between the relatively small Dacron graft and a very large aortic aneurysm.

In this report, we have described a novel technique of “*V*” type aortoplasty of the proximal descending aorta in order to decrease the aortic aneurysmal dimension and to facilitate the performing of anastomosis between the Dacron graft and aortic aneurysm.

## Conclusion

Patients with complex aortic pathology, especially with an aneurysm involving the aortic arch and proximal descending aorta, have been treated in one or two stages. In cases of very large aortic aneurysmal dimensions where the elephant trunk procedure is required, it might be technically difficult to perform anastomosis between a relatively small Dacron graft and a very wide aortic aneurysm. A surgical solution in such cases remains undefined and will likely need to be determined on a case-by-case basis. Our patient underwent complex aortic arch repair with the Dacron graft using a so-called island technique. In order to decrease the aortic aneurysmal dimension to facilitate the performance of anastomosis between the Dacron graft and aortic aneurysm, we successfully used the novel technique of “*V*” type aortoplasty of the proximal descending aorta. Our surgical approach was dictated by the patient’s anatomical features. The patient is still alive 20 months following the repair.

## Consent

Written informed consent was obtained from the patient for publication of this Case report and any accompanying images. A copy of the written consent is available for review by the Editor-in-Chief of this journal.

## Abbreviations

SLE: Systemic lupus erythematosus
STJ: Sinotubular junction
CT: Computerized tomography
TTE: Trans-thoracic echocardiographic
TEE: Trans-esophageal echocardiographic
AoR: Aortic regurgitation
EF: Ejection fraction
MR: Mitral regurgitation
TR: Tricuspid regurgitation
PAH: Pulmonary artery hypertension
PASP: Pulmonary artery systolic pressure
CNS: Central nervous system
PNS: Peripheral nervous system
SPECT: Single photon emission computed tomography
